# Comparing genomic studies in animal breeding and human genetics: focus on disease-related traits in livestock — A review

**DOI:** 10.5713/ab.24.0487

**Published:** 2024-10-24

**Authors:** Olivier Gervais, Yoshitaka Nagamine

**Affiliations:** 1 College of International Relations, Nihon University, Mishima, Shizuoka 411-8555, Japan; 2 Center for Genomic Medicine, Graduate School of Medicine, Kyoto University, Sakyoku, Kyoto 606-8507, Japan; 3 College of Bioresource Sciences, Nihon University, Fujisawa, Kanagawa 252-0880, Japan

**Keywords:** Disease Resistance, Genomics, Genome-wide Association Studies (GWAS), Livestock, Medical Genetics, Polygenic Models

## Abstract

Genomic studies of diseases can be divided into two types: i) analyses that reveal causal genes by focusing on linkage disequilibrium between observed and causal variants and ii) those that simultaneously assess numerous genetic markers to estimate the polygenic effects of a particular genomic region or entire genome. The field of human genetics has emphasized the discovery of causal genes, but these represent only a fraction of the total genetic variance. Therefore, alternative approaches, such as the polygenic risk score, which estimates the genetic risk for a given trait or disease based on all genetic markers (rather than on known causal variants only), have begun to garner attention. In many respects, these human genetic methods are similar to those originally developed for the estimation of breeding values (i.e., total additive genetic effects) in livestock. However, despite these similarities in methods, the fields of human and animal genetics still differ markedly in terms of research objectives, target populations, and other characteristics. For example, livestock populations have continually been selected and inbred throughout their history; consequently, their effective population size has shrunk and preferred genes (such as those influencing disease resistance and production traits) have accumulated in the modern breeding populations. By examining the characteristics of these two fields, particularly from the perspectives of disease and disease resistance, this review aims to improve understanding of the intrinsic differences between genomic studies using human compared with livestock populations.

## INTRODUCTION

The use of genomic information to improve livestock has accelerated over the past 10 to 15 years [1]. During this same period, the field of human genetics has identified numerous disease-related genes [2]. Although the goals of genomic studies differ markedly between human and animal populations (i.e., medical care vs commercial production), the differences in methodology between human and livestock genetics are relatively minor. In this review, by comparing efforts to improve disease resistance in livestock with research in human genetics, we hope to increase understanding of the intrinsic similarities and differences between these two fields.

## THE TWO TYPES OF GENOMIC STUDIES

Genomic studies of diseases are broadly divided into two types: i) analyses that detect causal genes through assessing linkage disequilibrium between observed and causal variants and ii) those that simultaneously evaluate multiple markers to estimate the polygenic effects of a given genetic region or entire genome. One example of the first type is a 2001 landmark publication in the field of animal breeding [3], which proposed using densely mapped markers to identify quantitative trait loci (QTLs) throughout the genome; in turn, the total genetic value of the discovered QTLs would be applied as an estimated breeding value for genomic selection. However, identifying all the QTLs for a given trait is incredibly challenging, even in the medical field with its vast resources (e.g., funding, datasets), and this limitation has led to the problem of ‘missing heritability’ [4].

However, single-nucleotide polymorphisms (SNPs) significant at the genome level are insufficient to explain the total heritability of a specific trait. Therefore, methods for the second type of genomic study were developed [5,6]; these incorporate all available SNPs to estimate the total polygenic effect. By dividing chromosomes into smaller units containing of a few tens or hundreds of SNPs, the total polygenic effect of a given genomic region can be estimated [7].

These SNP-based methods stem from the field of animal breeding, namely from Henderson’s best linear unbiased predictor (BLUP) model [8]. Such methods have been applied effectively to both livestock [9] and human [10] populations, leading to the development of additional methods as well as software for their implementation [11]. For example, instead of following the traditional BLUP model, which is built from a numerator relationship matrix (A) calculated from pedigree information (i.e., the genetic relationships between individuals), current BLUP-type methods typically involve a genomic relationship matrix (G) [12–16] computed from numerous markers (i.e., SNPs) spread across the target species’ genome. Methods developed subsequent to Henderson’s BLUP include the single-step genomic BLUP [17,18], which combines a pedigree-based A matrix with a marker-based G matrix for improved efficiency.

Although animal genetics has historically emphasized polygenic effects and the estimation of breeding values for genomic selection, the identification of causal genes underlying a variety of traits is also a major goal in livestock. This includes many important Mendelian diseases to select against, such as arachnomelia (i.e., a disease characterized by abnormal skeletal development) and mulefoot disease (i.e., a disorder that results in fused hooves) [19].

Conversely, even though human genetics focuses to a great extent on single-SNP association methods, which test the effect of one SNP at a time, more complex statistical models—notably linear mixed models [10]—have been developed to address their limitations (e.g., the inclusion of related individuals). The results of these more sophisticated methods can in turn be used in other models, for instance as polygenic risk scores (PRS) for risk prediction in human diseases.

## THE UNDERLYING PRINCIPLES OF GENOME-WIDE ASSOCIATION STUDIES FOR GENE DISCOVERY

The general goal of genome-wide association studies (GWAS)—establishing the statistical correlation between genetic markers and causal genes (i.e., linkage disequilibrium)—has remained fundamentally unchanged since the earliest QTL analyses during the early 2000s [20–23]. However, the technologic evolution since the time when GWAS analyses were conducted by using only a few hundred markers (e.g., microsatellites) until now, when hundreds of thousands of markers (typically SNPs) densely distributed across the genome are involved, underlies the continued usefulness and success of GWAS. In other words, the dramatic increase in marker density has bolstered the usefulness of linkage disequilibrium between markers and causal variants, leading to key discoveries with noteworthy implications, particularly in the medical field.

Except in rare cases (e.g., intentional crossbreeding of animals to improve a specific trait), identifying the specific historical conditions that drove the linkage disequilibrium between a given marker and a causal variant (i.e., mutation, genetic drift) is difficult. Yet it is this very phenomenon of linkage disequilibrium itself that makes GWAS methods so effective for identifying causal variants in the first place.

To better understand the concept of linkage (dis)equilibrium, take the example of a normal gene X0, in which mutation X1 occurs, with marker C1 nearby. At first, X1 and C1 behave as though they are one and the same genetic moiety. That is, X1 and C1 display linkage disequilibrium—they are inherited nonrandomly. However, after several generations, recombination gives rise to instances where C1 is inherited with X0 rather than X1; eventually, X1 and C1 behave as though they are unrelated. That is, X1 and C1 are in a state of linkage equilibrium, such that C1 loses its usefulness as a marker for X1.

The rate of decline in linkage disequilibrium is given by


$${\left(1-\text{r}\right)}^{\text{t}},$$

where r is the recombination rate under random mating conditions, and t the number of generations [24]. An initial linkage disequilibrium of 100% (i.e., perfect correlation) would require 22 generations at r = 0.1 and 230 generations at r = 0.01 for linkage disequilibrium to decrease to 10%. Given that the recombination rate is proportional to the distance between the loci, the distance between X and C must be sufficiently small to prevent recombination (thereby keeping r low) and to maintain a state of linkage disequilibrium. Given that the location of a causal variant is unknown, to increase the likelihood of having a maker nearby, the overall number of markers must be increased dramatically. To increase the number of SNPs when resources are limited or to analyze a dataset with multiple sets of SNPs simultaneously, the method of imputation—the statistical inference of unknown genotypes—can be used. By using a reference panel of higher SNP density, imputation estimates the genotypes of unobserved variants between the available markers in the study population. Imputation is dependent on linkage disequilibrium.

For example, when the reference panel contains the genotype information of three SNPs as A-B-C but the known haplotype of the study population is A1-?-C1 (i.e., the information for B is missing), the number of markers can be increased by inferring (imputing) B. If the only haplotypes in the reference panel are A1-B1-C1 and A2-B2-C2, the missing genotype in A1-?-C1 can be inferred as B1 when recombination is rare. In cases with frequent recombination, the correlation between the loci must be estimated to infer the missing genotype; this process entails some degree of error. The accuracy of imputation is therefore dependent on the strength of the linkage disequilibrium in a given region and on the genetic similarity of that region between the two populations (i.e., the study population and the reference panel) [25,26].

**Figure 1:**
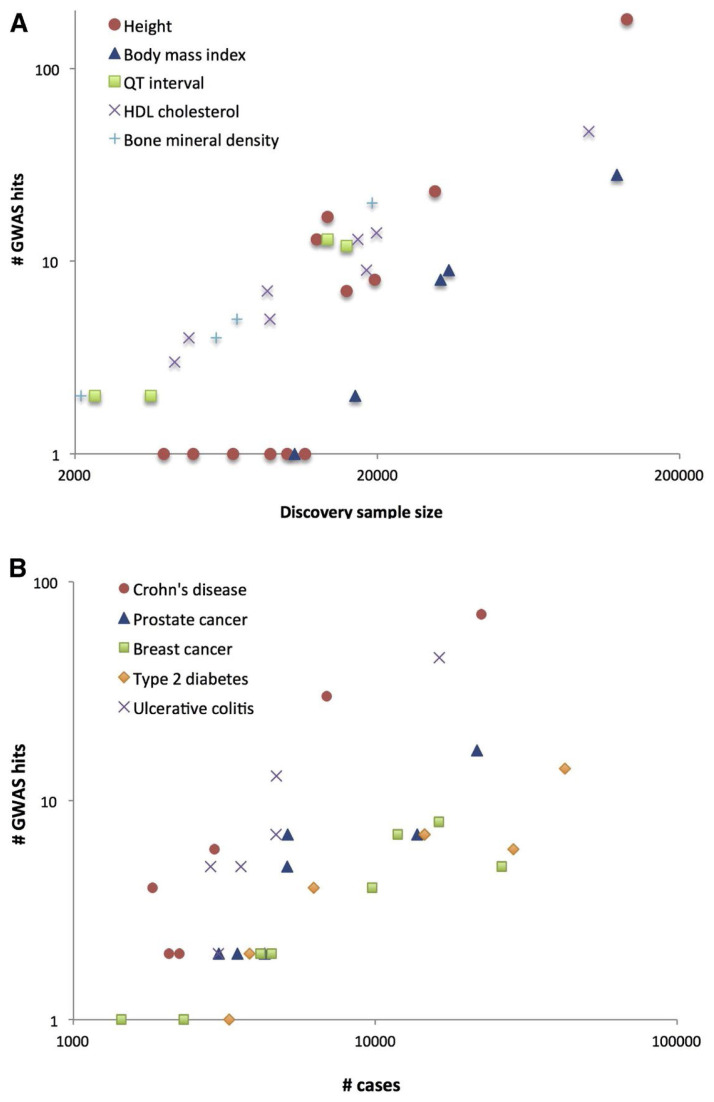
Increase in number of loci identified as a function of experimental sample size (Reprinted from Visscher et al [29] with permission of Elsevier.). (A) Selected quantitative traits. (B) Selected diseases.

If, for the sake of discussion, the aim of genomic studies is limited to using markers for the discovery of causal variants, the non-centrality parameter (NCP) can be applied to calculate the statistical power to detect an association from the correlation between an observed genotype (marker) and an unobserved causal variant [27,28], such that a higher NCP means higher statistical power:


$$\text{NCP}=\text{n}\times {\text{r}}^{2}\times {\text{q}}^{2}/(1-{\text{r}}^{2}{\text{q}}^{2}),$$

where n is the sample size, r^2^ is the squared correlation between the marker and the causal variant (i.e., the linkage disequilibrium), and q^2^ is the proportion of phenotypic variance explained by the causal variant. Therefore, r^2^q^2^ represents the proportion of phenotypic variance in the population that the marker accounts for, and the denominator (1–r^2^q^2^) is the proportion of phenotypic variance that the marker does not explain. This equation implies that a i) larger dataset, ii) stronger correlation between the marker and causal variant (i.e., linkage disequilibrium), and iii) greater effect of the causal variant are factors that translate into a larger numerator, therefore leading to increased detection power. In the extreme case in which the marker and causal variant share the same loci (e.g., whole-genome sequencing), r^2^ = 1, and r^2^q^2^ = q^2^. In that case,


$$\text{NCP}=\text{n}\times {\text{q}}^{2}/(1-{\text{q}}^{2}),$$

meaning that only the sample size and the proportion of phenotypic variance explained by the causal variant become relevant. This situation shows the importance of sample size and marker density to increase the statistical power of genomic studies [29] (Figure 1).

Furthermore, this equation is influenced by the allele frequency of the causal variant (F), according to the following equation [28]:


$${\text{q}}^{2}=2\times \text{F}\times (1-\text{F})\times \beta^{2},$$

where β is the effect size on the phenotype of the causal variant (in standard deviation units). The proportion of phenotypic variance explained by the causal variant, q^2^, is highest at F = 0.5 and lowest at F = 0 or 1. This explains why rare variants (i.e., F is close to 0) are extremely difficult to detect, despite making a disproportionate contribution to phenotypic variation due to their larger effect sizes [30], for instance in the case of loss-of-function variants (i.e., variants which prevent the generation of functional proteins). Indeed, although rare variants play a significant role in human diseases—including many Mendelian disorders—, very large sample sizes or effect sizes are required to achieve adequate statistical power [31]. All things considered, the only factors that can be adjusted are sample size (n) and the number of markers (the more SNPs, the higher r^2^). Figure 1 [29] illustrates the relationship between the number of loci identified by using GWAS in the medical field and the sample size (A) or number of cases (B). In line with the definition of the NCP, it shows empirically that in GWAS the number of detected variants is strongly correlated with sample size, and that a doubling in sample size leads approximately to a doubling in the number of detected variants.

## OVERVIEW OF GENOMIC STUDIES IN HUMAN MEDICINE

The rapid development of DNA sequencing technology was built on the invention of the first automated DNA sequencer in the mid-1980s and was essential to the completion in 2003 of the Human Genome Project, a 13-year international effort representing a US$3 billion investment [32]. The first GWAS study in humans was published in 2002 [33], and international consortia since have expended considerable economic and human resources to further this development. As a result, the GWAS Catalog (https://www.ebi.ac.uk/gwas/), an extensive database of SNP–trait associations, is the compilation of more than 6,000 publications and has contributed to the identification of approximately 400,000 associations across thousands of human traits [34]. In addition, human GWAS studies have led to the development of several new drugs for type 2 diabetes and hyperlipidemia, among other diseases [35,36]. In addition, genetic testing is becoming increasingly common worldwide, thereby facilitating the diagnosis and treatment of rare diseases [37]. Although ethical issues remain regarding the use of genetic testing, this trend is likely to continue in the future. These successes highlight the usefulness of identifying new susceptibility genes in furthering our understanding of the underlying causes of human diseases and in achieving new treatments.

The human genome (3.1 billion base pairs [Gb]) is similar in size to those of cows (2.9 Gb) and pigs (2.7 Gb), and GWAS methodology differs little between the fields of human genetics and animal breeding. However, the absence of concepts such as breeding value and selection in human genetics highlights the fundamental differences in terms of the aims and limitations of these fields. Human medical genetics has focused on investigating underlying etiology, drug discovery, and treatments tailored to the needs of individual patients. Consequently, considerable emphasis has been placed on identifying new genes for various diseases and related traits, primarily through improving the accuracy of SNP testing.

Even with all the resources available in the medical field, the susceptibility genes detected through increasing SNP accuracy account for only a small portion of a phenotype’s heritability [4], leading to the ‘missing heritability’ problem. However, this issue was largely resolved by incorporating all sequence variants from whole-genome sequencing data, including rare variants, in heritability estimation [5,6,38].

Methods that estimate the total polygenic effect—i.e., that incorporate all available markers and not only those that reach the significance threshold in GWAS—have garnered attention in human genetics recently. This trend is driven primarily by an increasing interest in predicting human diseases through the calculation of PRS. These calculations incorporate all known (i.e., including non-statistically significant) genetic markers to estimate a person’s risk of developing a given disease or condition [6,39,40]. Most PRS studies to date have focused on common variants (i.e., MAF >1%), but the addition of rare variants to PRS models is known to substantially increase prediction accuracy in carriers [41]. Some PRS models now account for rare variants to better predict breast cancer risk [42,43].

Because PRS focuses on the total polygenic effect to assess risk, its use of additional variants to those known to be associated with the target disease renders it equivalent to the concept of breeding value in livestock genetics. Breeding value is an estimate of an animal’s genetic merit that cannot be determined only from phenotypic information. Several methods developed for the estimation of total polygenic effect in animal breeding (i.e., breeding value), such as G-BLUP, have also been implemented in human genetics [44].

Given the similarities of these methods, similar issues tend to arise with their use in both animal and human genetics. For example, although it is possible to accurately estimate the breeding value of a given individual in a training population for which the association between genotype and phenotype is known, the accuracy will drop substantially when trying to estimate the breeding values of individuals in a target population that is genetically different [45]. The same phenomenon has been reported for PRS in humans: using a European training population to estimate the PRS in a non-European target population yields estimates of low accuracy [46].

Differences also exist, notably due to the intrinsic characteristics of humans and livestock. For instance, inter-individual environmental differences (e.g., differences in diet or treatment) are more pronounced in humans. Another difference is that PRS is used to predict the future phenotype of a given individual at a clinical level, whereas in livestock estimated breeding values are used to select the animals with the best genetic potential as parents for the next generation, with the aim to improve the mean breeding value over time [47]. Results are therefore interpreted differently. In addition, in species like cattle some animals (e.g., bulls) produce a great number of offspring. Their genetic merit can therefore be evaluated very precisely, which is rarely the case in humans.

## GWAS FOR HUMAN DISEASES COMPARED WITH GWAS FOR DISEASE-RESISTANCE TRAITS IN LIVESTOCK

When comparing genomic studies in humans with those in livestock, it is first necessary to be aware of the differences that exist in terms of perspective. For example, in human genetics, the concept of penetrance is commonly used to describe the expression of the disease phenotype (e.g., “in individuals with the same genotype, the onset of disease is contingent on penetrance”). By contrast, in animal breeding, the expression (or not) of disease typically is treated as a threshold trait, the underlying breeding value of which is based on an assumed continuous distribution (usually normal distribution) of factors contributing to the trait. As such, the higher the breeding value of susceptibility (i.e., the lower the breeding value of disease resistance), the higher the risk of developing the disease. In simpler terms, human genetics tends to focus on whether the genes are expressed to determine the phenotype, whereas animal genetics primarily addresses the issue as the sum of the additive genetic effects of a quantitative trait, as embodied by the term ‘breeding value.’

Unsurprisingly, this fundamental difference is reflected in the genomic studies in these fields. In the human medical field, in spite of the increasing use of complex statistical models like linear mixed models, most efforts are dedicated to the discovery of new, singular genes, whereas genetic studies benefitting animal breeding tend to focus on the effects of undetectable polygenes as a matter of course. Incidentally, the term ‘complex trait’ as used in human genetics often refers to what is called ‘quantitative trait’ in animal breeding. In many cases, the term ‘complex trait’ is used to refer to any trait that is not driven by a single gene, regardless of whether the outcome is discrete or continuous [48,49]. Given that the term ‘quantitative trait’ is only used to refer to traits for which phenotypic variation is continuously distributed, these terms are equivalent when referring to non-Mendelian traits with a quantitative outcome. However, exceptions do exist [29]. In that article, the author, whose background is in animal breeding, makes a terminological distinction between quantitative traits (continuous variables; Figure 1A) and complex traits (discrete variables, indicating the presence or absence of disease; Figure 1B).

In addition to these distinct viewpoints, several important differences need to be considered between genomic studies on human diseases in the medical field and disease-resistance traits in animal breeding. First, compared with human medicine, the field of animal breeding is at a disadvantage due to i) insufficient financial and human resources and ii) incomplete and limited detailed information regarding the cause of death of sick animals. The first point is self-explanatory, but the second is likewise critical for understanding the difficulty in improving disease resistance in livestock. Although the low heritability of disease resistance traits does play a role, the lack of precise information on the medical condition of livestock greatly impedes the study of disease resistance. One such illustration is a study on mycoplasmal pneumonia in pigs [50]. The authors succeeded in improving the animals’ disease resistance by selecting for both disease resistance and meat production traits for five generations. As part of their selection index, the authors used mycoplasmal pneumonia scores based on direct measurements of lesions in sibling pigs, rather than simply using the presence or absence of disease [50].

Another example is a GWAS analysis of resistance to bovine tuberculosis in dairy cattle [51]. To identify false-positive tuberculin results, cattle that tested tuberculin-positive also underwent meat inspection, histopathology, and bacterial culture. These additional measures enabled the authors to explain 21% of the phenotypic variance in tuberculosis resistance [51].

In both example cases, these achievements would not have been possible without thorough measurements, suggesting the importance of detailed information on medical conditions for the improvement of disease resistance in livestock. When disease resistance is polygenic, an individual’s symptoms partly reflect its genetic potential. Therefore, precise knowledge about the extent of the condition is necessary to assess disease resistance appropriately.

The financial outlay and time needed to obtain genetic data for livestock have decreased dramatically in just a few years, thanks to the rapid development of sequencing technologies, and powerful analytical programs have become widely available. Nevertheless, despite the resulting increases in sample size and number of genetic markers, accurately assessing the genetic component of disease resistance remains challenging when phenotypes are not characterized accurately or completely. This lack of information becomes particularly conspicuous when comparing with the human medical field, where detailed medical records usually exist.

Conversely, conducting genetics research in livestock offers several advantages. First, the opportunity to use artificial hybridization and selection means that animals prone to different degrees of disease can be bred and studied. Second, the lower importance of identifying new treatment options for livestock is associated with a moderate impetus to discover new causal genes. Third, genetic research in livestock is associated with fewer constraints in terms of information protection and regulatory compliance, such as obtaining informed consent and managing incidental findings. A fourth benefit to animal genetics is its small effective population size. Several factors, including intense selection, inbreeding, skewed breeding sex ratio, and small population sizes are known give rise to relatively high levels of linkage disequilibrium [52,53]. This means that markers tag causal variants more efficiently, thus increasing the markers’ usefulness. Finally, compared with its human counterpart, the field of livestock genetics is less compelled to find causal genes that are relevant beyond a given genetic group. In human genetics, it is essential to identify genes that lead to the development of treatments that are applicable to the worldwide population. In contrast, selection in animals usually is applied within a given, localized population.

This last feature highlights a particularly important difference between these fields. In human genetics, GWAS studies typically are conducted in populations of unrelated individuals, to remove any bias that may occur due to familial relatedness. In addition, to avoid genetic and environmental biases stemming from differences between ancestral populations, human GWAS studies generally control for population stratification by using principal components analysis. These steps help filter out ‘noise,’ thereby facilitating the search for SNP–trait associations. In contrast, genomic studies in livestock typically include such bias (e.g., common environment, common causal genes within selected relatives) to some extent. However, this feature is not necessarily problematic, depending on the goals and set-up of the studies. For example, in a selection experiment on respiratory disease resistance involving five generations of pigs [50], SNP heritability was 20%, and a single significant QTL on chromosome 2 explained 87% of the genetic variance [54]. In addition, 21% of the phenotypic variance in a study on resistance to bovine tuberculosis [51] was explained by significant SNPs only, including 6.6% from chromosome 13. The sample sizes were 639 for the pig study [50] and 1,151 for the one in cattle [51]—far smaller than those of human genomic studies, which typically include thousands to tens of thousands of subjects (Figure 1B). In other words, the markers in the livestock studies adequately captured the genetic effects despite the small sample sizes. The fact that the experiments took place in populations that underwent selection for generations likely played a bigger role in the successful outcomes than did having well-defined phenotypes. Indeed, the detection of markers with large effects was likely facilitated by the accumulation of causal variants through selection. The existence of common environmental factors may also have contributed to the QTLs, explaining a large proportion of the phenotypic variation.

Selection for economic traits (including disease resistance) has been practiced for decades, successfully improving livestock populations. However, consequently, this process has led to considerable inbreeding, and livestock are—at least to some extent—subject to common environmental factors, such as feeding standards, rearing methods, and other factors at the local, regional, or national levels. For example, the average inbreeding coefficient of dairy cows in the United States already exceeds 8% [55].

In human genetics, although specific tools have been developed to account for related individuals (e.g., linear mixed models with pedigrees entirely specified), a conventional GWAS assumes that all individuals are unrelated [56]. This standard is meant to remove effects other than those directly stemming from causal variants, such as shared environmental factors and causal genes within relatives. Applying this human standard to livestock studies would remove individuals with high genetic merit from a population that is inevitably somewhat inbred and genetically relatively homogeneous. Given that the main objective in animal breeding is to efficiently improve the genetic potential of livestock through the selection of individuals of high genetic merit within a given population, changing the standards in place today to those similar to those currently in effect in the human field is unlikely to improve the effectiveness of breeding programs. In addition, because livestock populations that have undergone long-term selection are inbred and tend to be skewed genetically, some of the markers detected in a given livestock population may be useful only in the population in which they were identified and not in others.

## CONCLUSION

In this review, we have shown how differences between human compared with animal genetics studies—including study objectives and the structure of target populations—affect the results of those studies and their interpretation. Understanding the characteristics and caveats of each field provides insights into how similar methodology can successfully be applied toward achieving different aims. This awareness highlights the deep need for both human and animal geneticists to learn from each other’s field.
